# Filamin A interacting protein 1-like expression inhibits progression in colorectal cancer

**DOI:** 10.18632/oncotarget.12664

**Published:** 2016-10-14

**Authors:** Young-Lan Park, Sun-Young Park, Seung-Hyun Lee, Rul-Bin Kim, Joong-Keun Kim, Sung-Yoon Rew, Dae-Seong Myung, Sung-Bum Cho, Wan-Sik Lee, Hyun-Soo Kim, Young-Eun Joo

**Affiliations:** ^1^ Department of Internal Medicine, Chonnam National University Medical School, Gwangju, Republic of Korea

**Keywords:** FILIP1L, cell survival, angiogenesis, prognosis, colorectal neoplasm

## Abstract

Filamin A interacting protein 1-like (FILIP1L) expression, which is decreased in various cancers, may inhibit carcinogenesis. In this study, we evaluated the effects of FILIP1L on oncogenic behavior and prognosis in colorectal cancer. siRNA-mediated FILIP1L knockdown enhanced tumor cell migration and invasion and inhibited apoptosis and cell cycle arrest in COLO205 cells. pcDNA-myc vector-mediated FILIP1L overexpression suppressed tumor cell migration and invasion and induced apoptosis and cell cycle arrest in HCT116 cells. FILIP1L knockdown enhanced angiogenesis by increasing VEGF-A and HIF-1α levels and decreasing angiostatin level. FILIP1L overexpression suppressed angiogenesis by decreasing VEGF-A and -D l level and increasing angiostatin and endostatin levels. Phosphorylated β-catenin levels decreased and phosphorylated Akt and GSK-3β levels increased following FILIP1L knockdown. FILIP1L overexpression had the opposite effects. FILIP1L expression was associated with reductions in tumor size, cell differentiation, lymphovascular invasion, stage, invasion depth and lymph node metastasis, and with longer overall survival. Mean Ki-67 labeling indexes and microvessel density values were lower in FILIP1L-positive tumors than in FILIP1L-negative tumors. These results indicate that FILIP1L suppresses tumor progression by inhibiting cell proliferation and angiogenesis in colorectal cancer.

## INTRODUCTION

Colorectal cancer is a leading cause of cancer-related morbidity and mortality worldwide. Despite recent advances in treatment, overall survival remains poor in advanced colorectal cancer patients [1–3]. Metastasis is a major cause of death in patients with a variety of cancers. Metastasis is a complex process resulting from multiple alterations in proto-oncogenes and tumor suppressor genes that promote growth, differentiation, migration, invasion, cell survival, and angiogenesis in cancer cells [4–6]. A better understanding of the pathobiology underlying colorectal cancer metastasis may aid in predicting cancer progression and provide new molecular targets for cancer therapy.

Treatment with angiogenic inhibitors up-regulated Filamin A interacting protein 1-like (FILIP1L) in a microarray study examining gene expression profiles in human umbilical vein endothelial cells (HUVECs) [7, 8]. FILIP1L may play a role in cytoskeletal remodeling and regulate cell polarity and motility [9–11]. FILIP1L expression in endothelial cells also inhibits cell proliferation and migration and increases apoptosis [12]. Targeted expression of truncated mutant FILIP1L decreases tumor volume by inhibiting tumor neovascularization and inducing apoptosis and necrosis in both human malignant melanoma tumor xenograft models and serous cystadenocarcinoma cells [12, 13]. In addition, FILIP1L mRNA and protein levels are down-regulated in various human cancers, including ovarian, prostate, breast, lung, pancreatic, and colorectal cancers [14–18]. These data indicate that FILIP1L may act as a tumor suppressor gene by decreasing cell proliferation, migration, and angiogenesis and increasing apoptosis [7–18]. Although FILIP1L may be a promising molecular target for cancer treatment, whether FILIP1L suppresses tumor progression in colorectal cancer remains unknown.

To evaluate whether FILIP1L affects growth and metastasis in colorectal cancer, we examined FILIP1L expression in human colorectal cancer tissues and its association with clinicopathological features, including overall survival.

## RESULTS

### FILIP1L expression in human colorectal cancer cell lines

**Figure 1:**
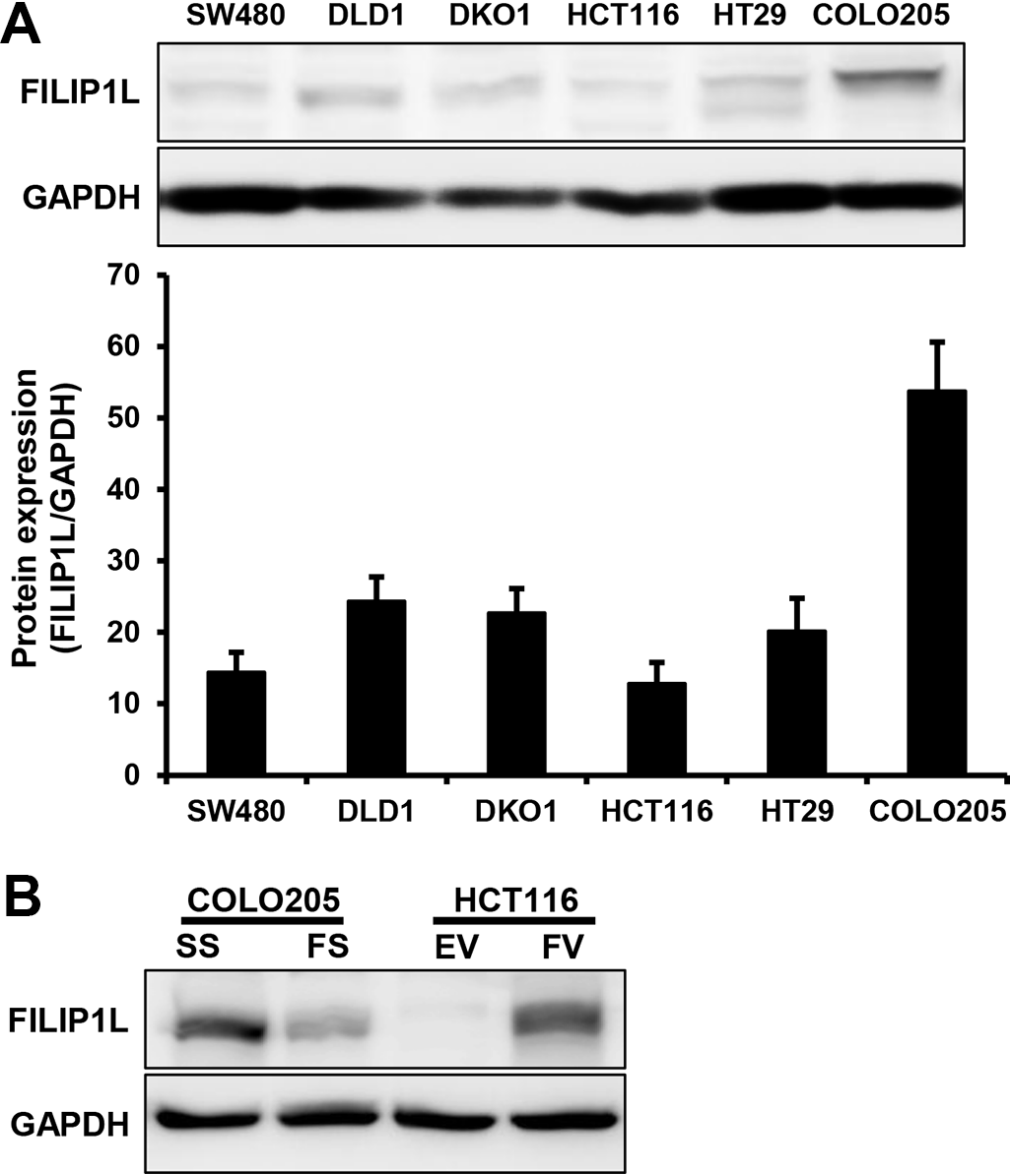
FILIP1L protein levels in human colorectal cancer cells (**A**). Endogenous FILIP1L protein levels were measured in various human colorectal cancer cell lines using Western blots. GAPDH was used as a loading control. Graphical representations of band intensities quantified using the Multi-Gauge Ver3.0 gel analysis software are shown. (**B**). Knockdown and overexpression of FILIP1L protein was performed using FILIP1L siRNA and pcDNA6-myc-FILIP1L, respectively. SS, scramble siRNA; FS, FILIP1L siRNA; EV, empty-pcDNA6-myc vector; FV, pcDNA6-myc-FILIP1L vector.

FILIP1L protein levels were examined by Western blotting in various human colorectal cancer cell lines, including SW480, DLD1, DKO1, HCT116, HT29, and COLO205 cells. Among these, FILIP1L protein levels were highest in COLO205 cells and lowest in HCT116 cells (Figure 1A). siRNA-mediated FILIP1L knockdown decreased protein levels in COLO205 cells, and pcDNA6-myc-FILIP1L vector transfection increased protein levels in HCT116 cells (Figure 1B).

### FILIP1L inhibits migration and invasion in human colorectal cancer cells

**Figure 2:**
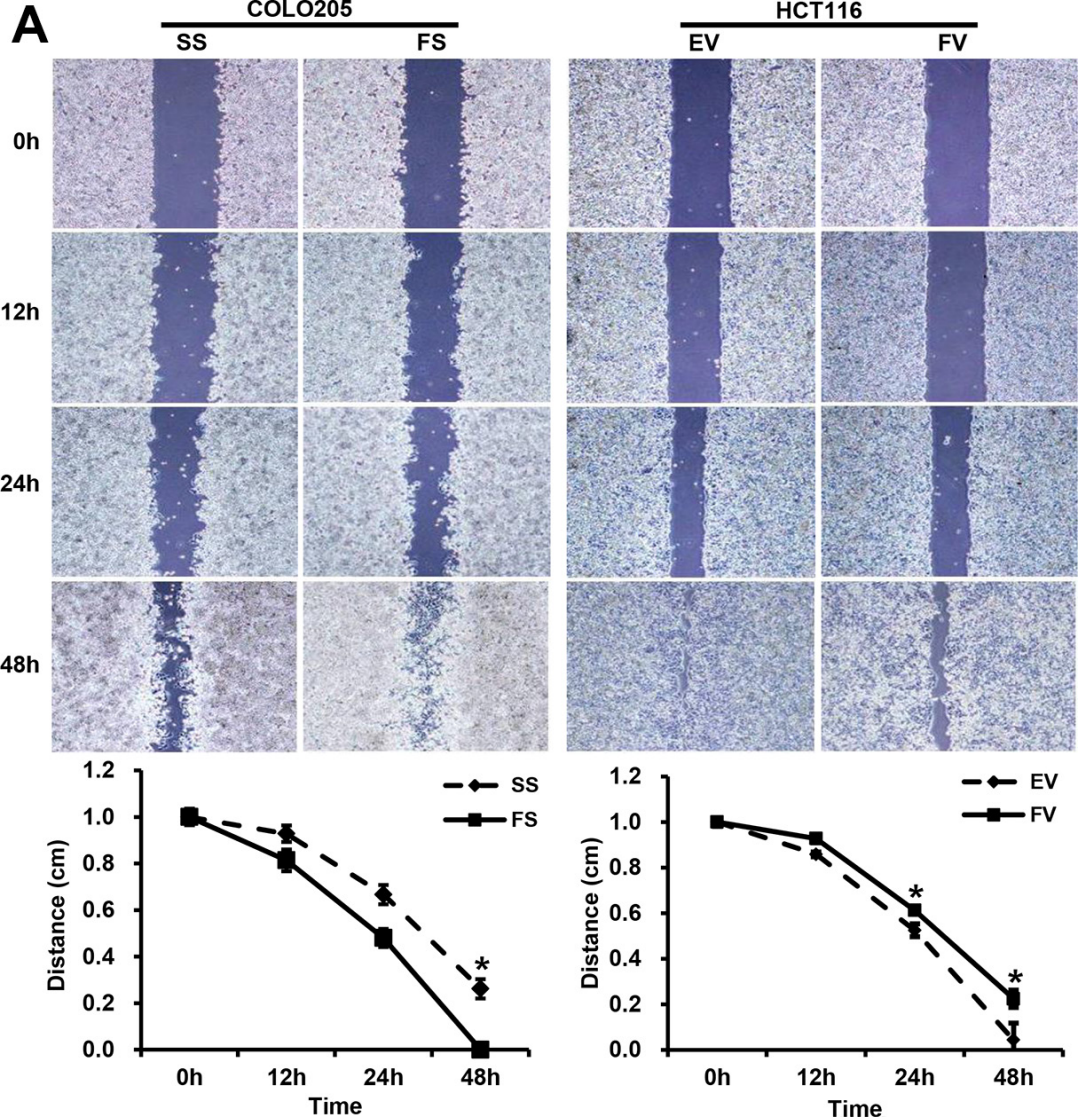

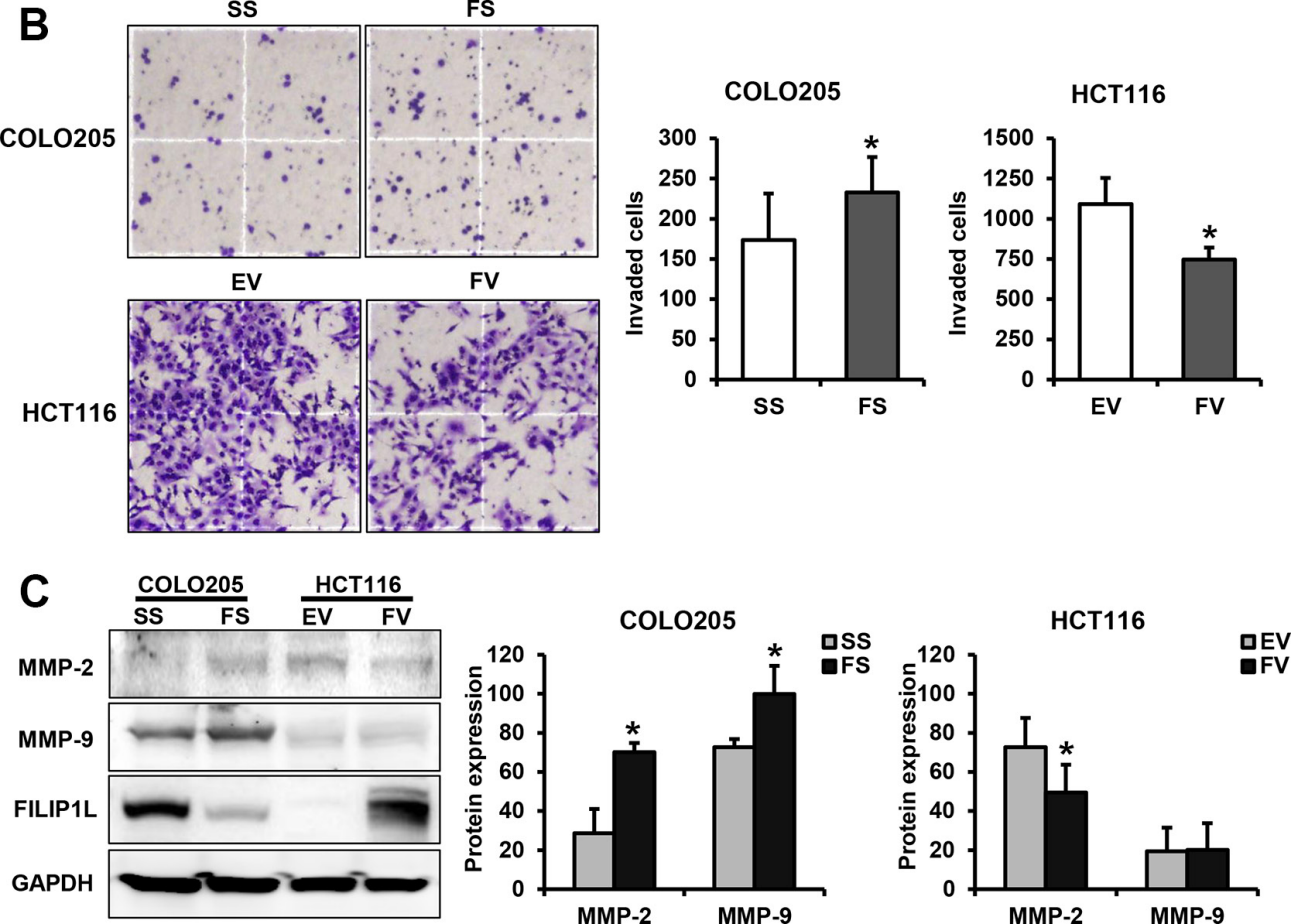
FILIP1L inhibits migration and invasion in human colorectal cancer cells (**A**) The impact of FILIP1L on colorectal cancer cell migration. Wound healing assays were performed using FILIP1L siRNA- or pcDNA6-myc vector-transfected cells; graphs of cell migration display relative healing distances (mean ± SE, *n* = 3; *P < 0.05). (**B**) The impact of FILIP1L on colorectal cancer cell invasion. The invasion assay was performed using FILIP1L siRNA- or pcDNA6-myc vector-transfected cells. Stained invading cells were counted and are shown for each group (mean ± SE, *n* = 6; *P < 0.05). (**C**) The impact of FILIP1L on MMP-2 and -9 levels in colorectal cancer cells. Graphs show the mean ± SE (*P < 0.05). SS, scramble siRNA; FS, FILIP1L siRNA; EV, empty-pcDNA6-myc vector; FV, pcDNA6-myc-FILIP1L vector.

FILIP1L siRNA transfection reduced wound gap sizes in plated COLO205 cells compared to scramble siRNA-transfected cells after 48 h (*P* = 0.008). Conversely, wound gap sizes increased in pcDNA6-myc-FILIP1L-transfected HCT116 cells compared to empty-pcDNA6-myc-transfected cells after 24 and 48 h (*P* = 0.033 and 0.030, respectively; Figure 2A). Similarly, FILIP1L siRNA transfection in COLO205 cells increased (*P* = 0.049), while pcDNA6-myc-FILIP1L transfection in HCT116 cells decreased (*P* = 0.004), numbers of invading cells compared to scramble siRNA transfection and empty-pcDNA6-myc transfection, respectively (Figure 2B). MMP-2 and -9 levels increased in COLO205 cells after FILIP1L knockdown (*P* = 0.047 and 0.045, respectively). In HCT116 cells overexpressing FILIP1L, MMP-2 level decreased, but MMP-9 level did not change (*P* = 0.001 and 0.545, respectively; Figure 2C).

### FILIP1L promotes apoptosis and cell cycle arrest in human colorectal cancer cells

**Figure 3:**
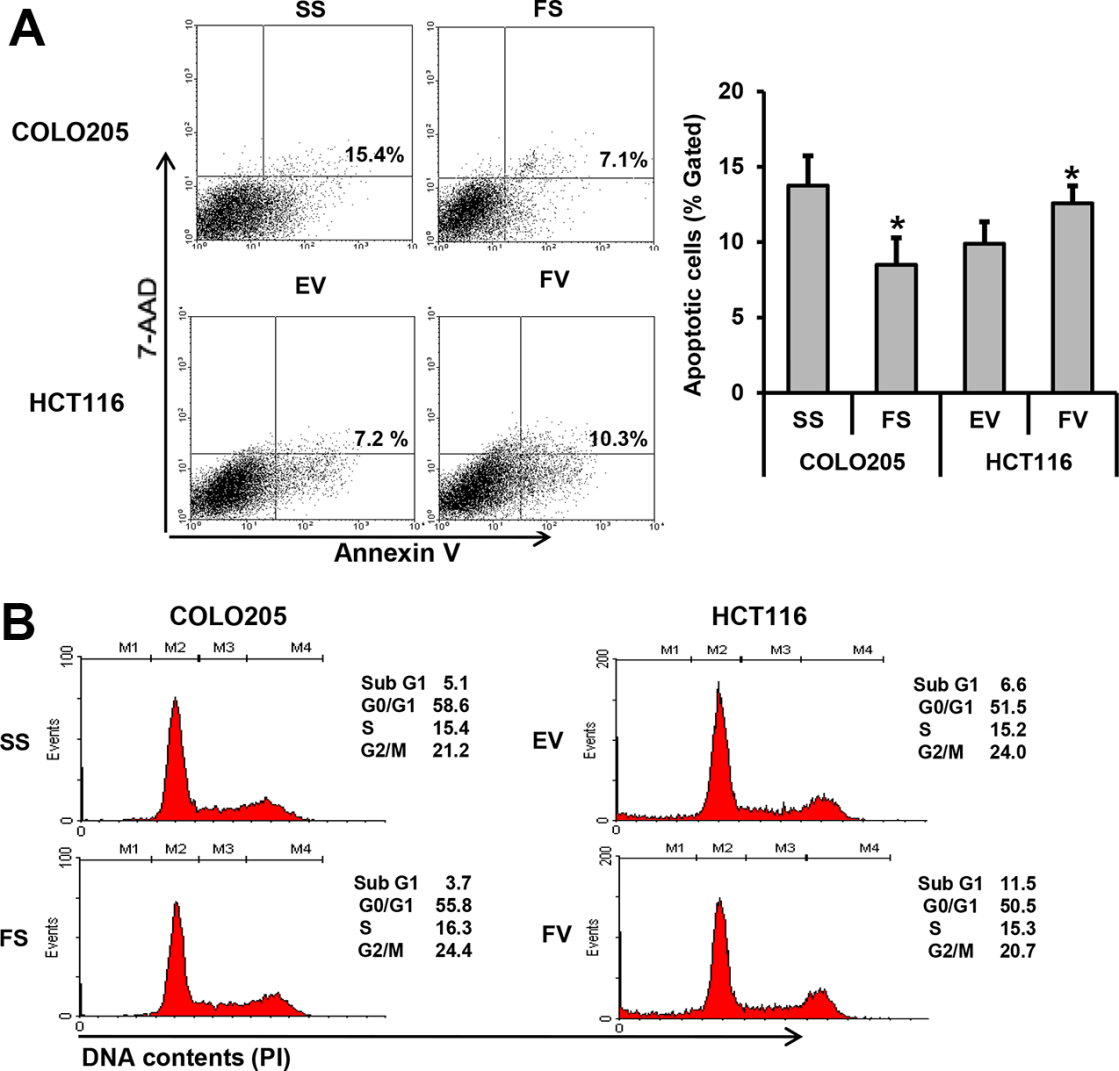

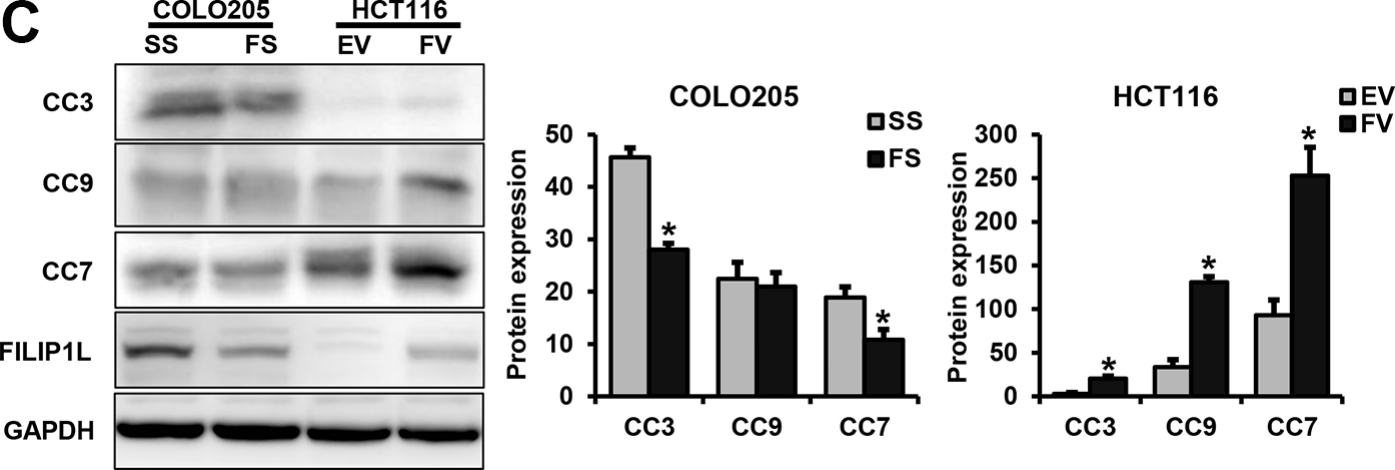
FILIP1L promotes apoptosis and cell cycle arrest in human colorectal cancer cells Flow cytometric analyses and Western blotting were performed to evaluate the impact of FILIP1L on apoptosis and cell cycle distribution. (**A**) FILIP1L promotes apoptosis (mean ± SE, *n* = 3; *P < 0.05). (**B**) FILIP1L promotes cell cycle arrest. (**C**) The impact of FILIP1L on cleaved caspase-3 (CC3), -9 (CC9) and -7 (CC7) levels. Graphs show the mean ± SE (*P < 0.05). SS, scrambled siRNA; FS, FILIP1L siRNA; EV, empty-pcDNA6-myc vector; FV, pcDNA6-myc-FILIP1L vector.

We then used flow cytometry to evaluate the impact of FILIP1L on apoptosis and cell cycle distribution. Apoptosis rates decreased after transfection with FILIP1L siRNA compared to transfection with scramble siRNA in COLO205 cells (15.4 *vs.* 7.1%, *P* = 0.014). In addition, apoptosis rates increased after overexpression of FILIP1L in HCT116 cells (7.2 *vs.* 10.3%, *P* = 0.047; Figure 3A). FILIP1L overexpression increased sub-G1 phase arrest in HCT116 cells, and FILIP1L knockdown decreased cell cycle arrest in COLO205 cells (Figure 3B).

Next, we measured FILIP1L knockdown- and overexpression-induced changes in caspase levels. Cleaved caspase-3 and -7 levels decreased in COLO205 cells after FILIP1L knockdown (*P* = 0.022 and 0.012, respectively). In contrast, cleaved caspase-3, -9, and -7 levels increased in HCT116 cells after FILIP1L overexpression (*P* = 0.005, 0.023, and 0.012, respectively; Figure 3C).

### FILIP1L inhibits angiogenesis in human colorectal cancer cells

**Figure 4:**
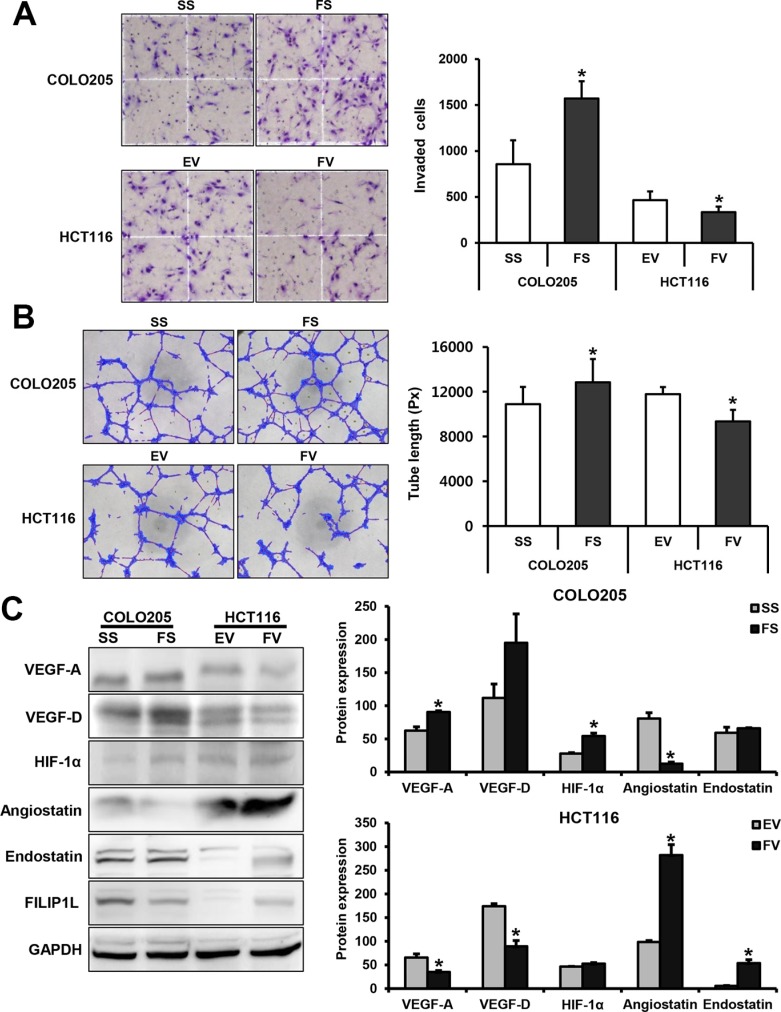
FILIP1L inhibits angiogenesis in human colorectal cancer cells Matrigel invasion and tube formation assays using conditioned medium from human colorectal cancer cells transfected with either FILIP1L siRNA or pcDNA6-myc vector were performed to evaluate the effects of FILIP1L on angiogenesis in human umbilical vein endothelial cells (HUVECs). (**A**) Invasion of HUVECs (mean ± SE, *n* = 3; *P < 0.05). (**B**) Tube formation in HUVECs (mean ± SE, *n* = 3; *P < 0.05). (**C**) The impact of FILIP1L on VEGF-A, -D, HIF-1α, angiostatin, and endostatin levels. Graphs show the mean ± SE (*P < 0.05). SS, scramble siRNA; FS, FILIP1L siRNA; EV, empty-pcDNA6-myc vector; FV, pcDNA6-myc-FILIP1L vector.

To evaluate the effects of FILIP1L on angiogenesis in HUVECs, we performed Matrigel invasion and tube formation assays using conditioned medium (CM) from human colorectal cancer cells transfected with either FILIP1L siRNA or pcDNA6-myc vector. Treatment with CM from FILIP1L siRNA-transfected COLO205 cells increased invasion in HUVECs compared to treatment with CM from scramble siRNA-transfected cells (*P* = 0.049). In contrast, treatment with CM from pcDNA6-myc-FILIP1L-transfected HCT116 cells decreased invasion in HUVECs compared to CM from empty-pcDNA6-myc-transfected cells (*P* = 0.004; Figure 4A). CM from FILIP1L siRNA-transfected COLO205 cells also increased endothelial tube formation in HUVECs compared to CM from scramble siRNA-transfected cells (*P* = 0.046). In contrast, treatment with CM from pcDNA6-myc-FILIP1L-transfected cells decreased tube formation in HUVECs compared to CM from empty-pcDNA6-myc-transfected cells (*P* = 0.003; Figure 4B). FILIP1L knockdown increased levels of the angiogenic inducers VEGF-A and HIF-1α in COLO205 cells (*P* = 0.020 and 0.026, respectively), while FILIP1L overexpression decreased VEGF-A and -D levels in HCT116 cells (*P* = 0.026 and 0.034, respectively). Moreover, FILIP1L knockdown decreased level of the angiogenic inhibitor angiostatin in COLO205 cells (*P* = 0.009), while FILIP1L overexpression increased levels of both angiostatin and endostatin in HCT116 cells (*P* = 0.017 and 0.027, respectively; Figure 4C).

### FILIP1L alters oncogenic signaling pathway activity in human colorectal cancer cells

**Figure 5:**
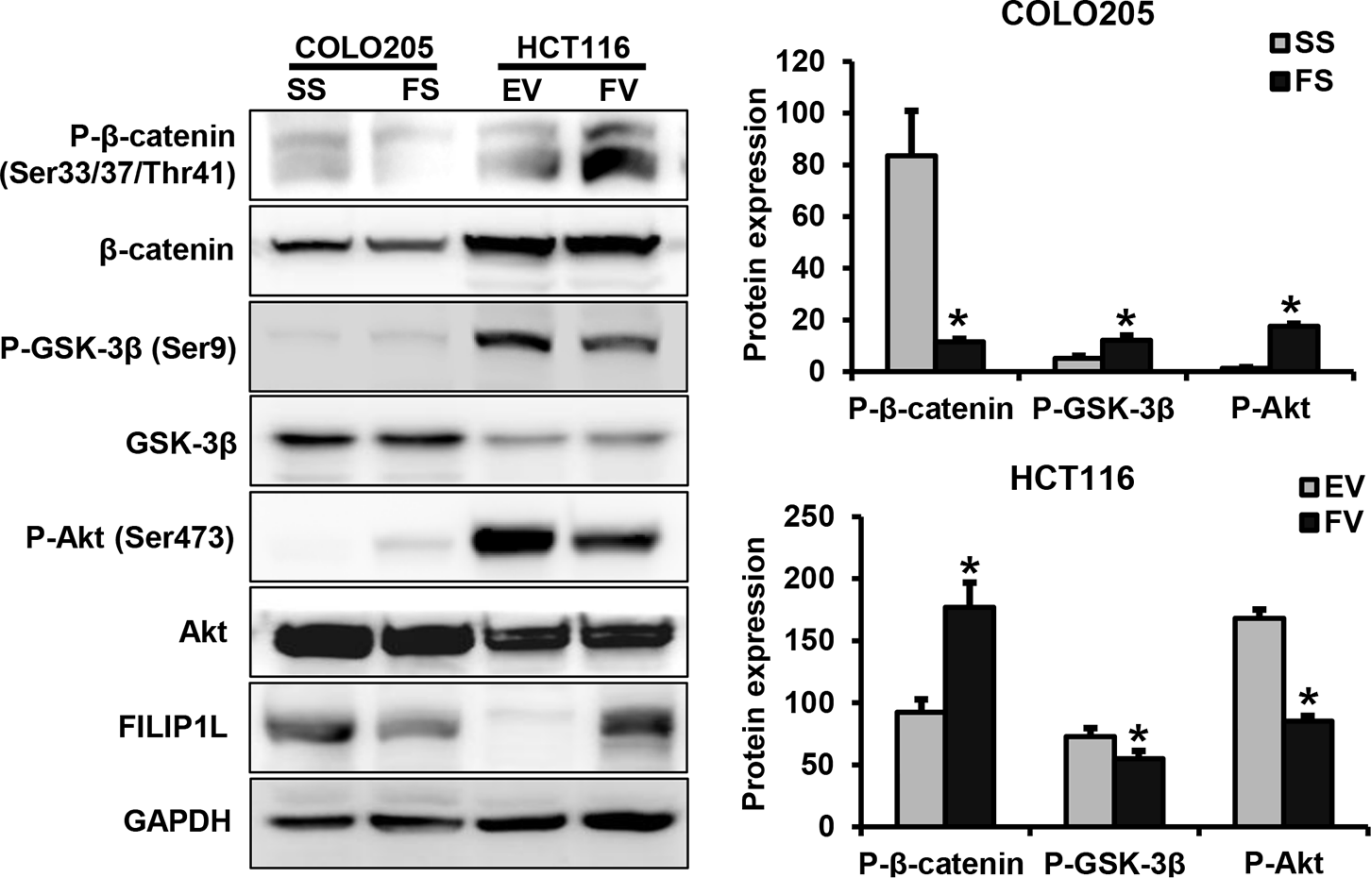
The impact of FILIP1L on intracellular signaling pathways in human colorectal cancer cells FILIP1L knockdown increased levels of phosphorylated Akt and GSK-3β and decreased level of phosphorylated β-catenin. In contrast, FILIP1L overexpression decreased levels of phosphorylated Akt and GSK-3β and increased level of phosphorylated β-catenin. Graphs show the mean ± SE (*P < 0.05). SS, scramble siRNA; FS, FILIP1L siRNA; EV, empty-pcDNA6-myc vector; FV, pcDNA6-myc-FILIP1L vector.

To examine whether FILIP1L activates oncogenic signaling pathways in human colorectal cancer cells, we measured phosphorylated β-catenin, Akt, and GSK-3β protein levels, using Western blotting. FILIP1L knockdown decreased phosphorylated β-catenin and increased phosphorylated Akt and GSK-3β levels in COLO205 cells (*P* = 0.047, 0.049, and 0.001, respectively). In contrast, FILIP1L overexpression increased phosphorylated β-catenin and decreased phosphorylated Akt and GSK-3β levels in HCT116 cells (*P* = 0.026, 0.003, and 0.008, respectively; Figure 5).

### FILIP1L was associated with clinicopathological parameters in human colorectal cancer

**Figure 6:**
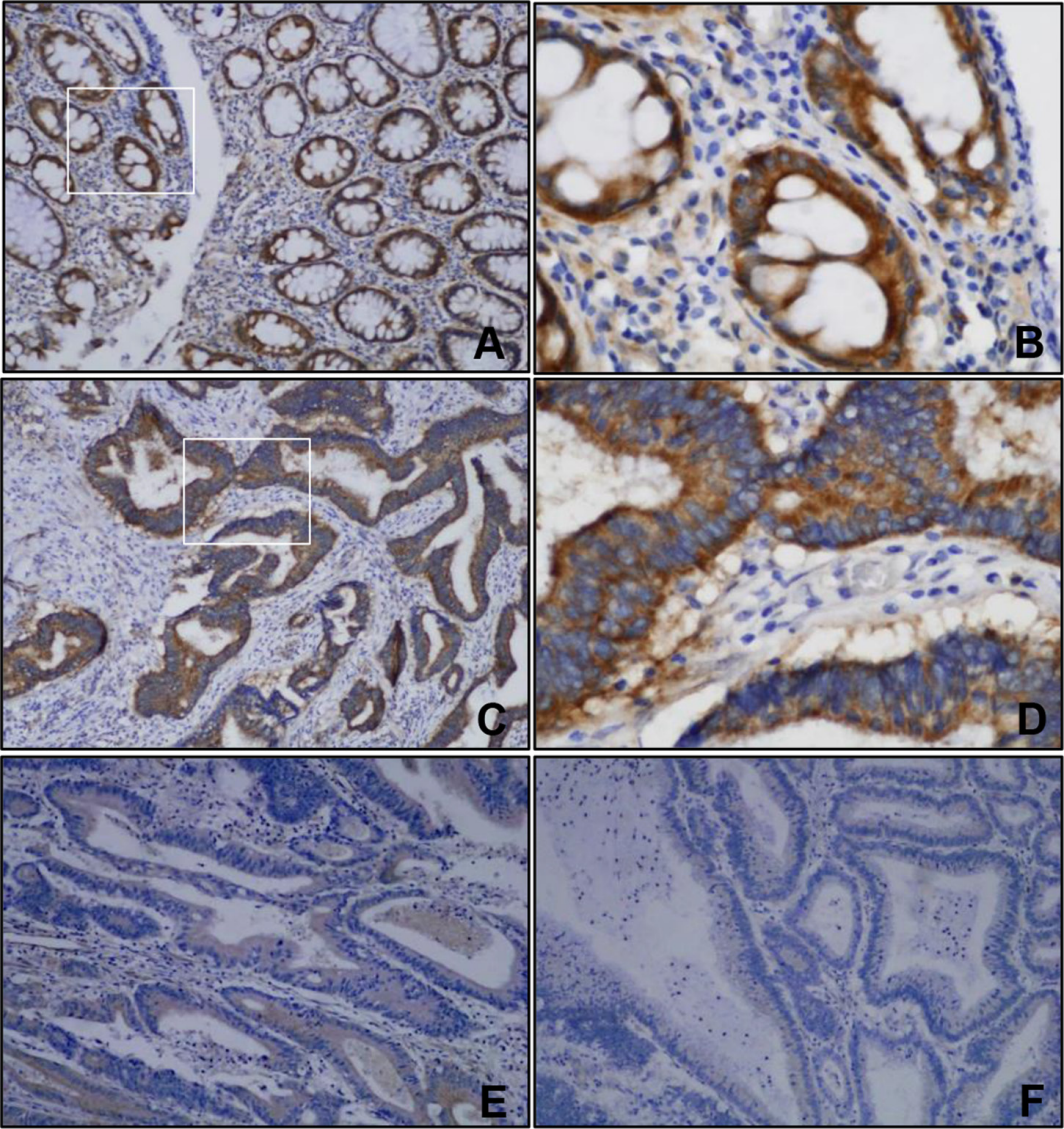
Representative images of immunohistochemical staining of FILIP1L (**A**) (×100) and (**B**) (×400), Strong FILIP1L expression was observed in the cytoplasm of normal colorectal epithelial cells. (**C**–**F**) FILIP1L expression in colorectal cancer tissues. (C) (×100) and (D) (×400), A score of 3 indicated strong immunostaining intensity for FILIP1L. (E) (×100), A score of 2 indicated moderate immunostaining intensity for FILIP1L. (F) (×100) A score of 1 indicated weak immunostaining intensity for FILIP1L.

**Figure 7:**
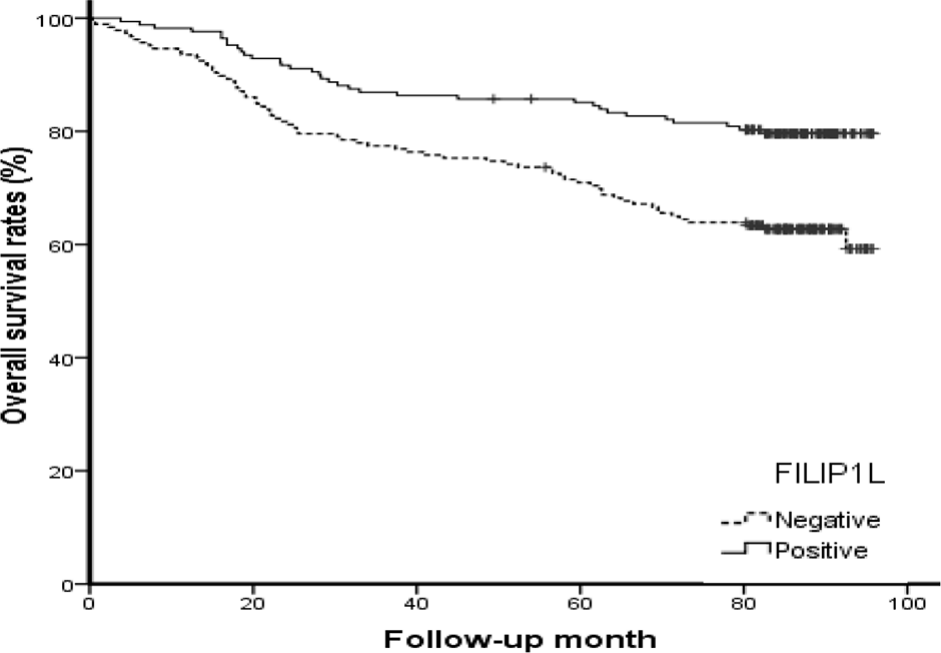
Kaplan-Meier survival curve showing the association between overall survival and positive (solid line) or negative (dotted line) FILIP1L expression

To determine the prognostic value of FILIP1L in human colorectal cancer, we examined associations between FILIP1L levels in formalin-fixed, paraffin-embedded tissue sections obtained from 354 colorectal cancer patients and clinicopathological data. Immunohistochemical FILIP1L protein staining was primarily cytoplasmic in normal and colorectal cancer cells (Figure 6A–6D). FILIP1L levels were lower in colorectal cancer cells than in normal colorectal epithelial cells (Figure 6E, 6F). Furthermore, higher FILIP1L levels were associated with reductions in tumor size, cell differentiation, lymphovascular invasion, cancer stage, depth of invasion, and lymph node metastasis (*P* = 0.019, < 0.001, 0.001, < 0.001, 0.005, and 0.002, respectively; Table 1). Moreover, patients with FILIP1L-positive tumors had longer overall survival than patients with FILIP1L-negative tumors (*P* < 0.001; Figure 7). To evaluate potential prognostic variables in colorectal cancer patients, multivariate analysis using the Cox proportional hazard model was performed. Age, tumor size, lymphovascular invasion, perineural invasion, and cancer stage were associated with survival. Negative FILIP1L expression was independently associated with poor overall survival after adjustment for several covariates, such as age, sex, tumor size, lymphovascular invasion, perineural invasion, and cancer stage (HR: 1.62; 95% CI: 1.07–2.46; *P* = 0.023; Table 2).

**Table 1:** Association between FILIP1L expression and clinicopathological parameters in human colorectal cancer patients

Parameters	Total (*n* = 354)	FILIP1L		*P*-value
		Negative (*n* = 186)	Positive (*n* = 168)	
Age (years)				0.650
< 69.8	135	73	62	
≥ 69.8	219	113	106	
Sex				0.247
Male	210	105	105	
Female	144	81	63	
Tumor size (cm)				0.019
< 4.9	194	91	103	
≥ 4.9	160	95	65	
Histologic type				< 0.001
Differentiated	313	152	161	
Undifferentiated	41	34	7	
Lymphovascular invasion				0.001
Negative	250	117	133	
Positive	104	69	35	
Perineural invasion				0.071
Negative	247	122	125	
Positive	107	64	43	
Stage				< 0.001
I/II	182	79	103	
III/IV	172	107	65	
Depth of invasion (T)				0.005
T1/T2	78	30	48	
T3/T4	276	156	120	
Lymph node metastasis (N)				0.002
N0	189	85	104	
N1–3	165	101	64	
Distant metastasis (M)				0.452
M0	309	160	149	
M1	45	26	19	

FILIP1L, Filamin A interacting protein 1-like.

**Table 2:** Cox multivariate regression of the association between FILIP1L expression and survival in colorectal cancer patients adjusted for clinicopathological parameters

Covariate	HR	(95% CI)	*P*-value
FILIP1L expression			
Positive	1.00		
Negative	1.62	1.07–2.46	0.023
Age			
< 69.8	1.00		
≥ 69.8	1.82	1.19–2.79	0.006
Sex			
Male	1.00		
Female	0.70	0.47–1.05	0.083
Tumor size			
< 4.9	1.00		
≥ 4.9	1.49	1.07–2.46	0.047
Lymphovascular invasion			
Negative	1.00		
Positive	1.62	1.18–2.81	0.023
Stage			
I/II	1.00		
III/IV	1.87	1.19–2.93	0.006
Perineural invasion			
Negative	1.00		
Positive	1.93	1.27–2.93	0.002

FILIP1L, Filamin A interacting protein 1-like; HR, Hazard ratio; CI, Confidence.

### Associations between FILIP1L expression and apoptosis, cell proliferation, and angiogenesis in human colorectal cancer

TUNEL assays and immunohistochemical staining of Ki-67 and CD34 were used to assess apoptosis, cell proliferation, and angiogenesis in all tumor cell samples. Apoptosis indexes (AI) in the 354 tumor samples ranged from 1.7 to 30.0, with a mean of 8.6 ± 5.9. AI did not differ depending on FILIP1L expression (*P* = 0.725). Ki-67 labeling indexes (KI) in the 354 tumor samples ranged from 21.9 to 89.3, with a mean of 59.4 ± 17.4. The mean KI value of 50.2 ± 13.1 in FILIP1L-positive tumors was lower than that in FILIP1L-negative tumors (*P* = 0.001). Microvessel density values (MVD) in the 354 tumor samples ranged from 14.0 to 151.0, with a mean of 65.6 ± 27.6. The mean MVD value of 58.3 ± 19.2 in FILIP1L-positive tumors was lower than that in FILIP1L-negative tumors (*P* = 0.020; Table 3).

**Table 3:** Associations between FILIP1L expression and apoptosis, proliferation, and angiogenesis in human colorectal cancer

Parameters (Mean ± SD)	Total (*n* = 354)	FILIP1L expression		*P*-value
		Negative (*n* = 186)	Positive (*n* = 168)	
AI	8.6 ± 5.9	8.2 ± 5.0	8.3 ± 6.8	0.725
KI	59.4 ± 17.4	66.1 ± 17.4	50.2 ± 13.1	0.001
MVD	65.6 ± 27.6	74.0 ± 33.8	58.3 ± 19.2	0.020

FILIP1L, Filamin A interacting protein 1-like; SD, Standard deviation; AI, Apoptotic index; KI, Ki-67 labeling index; MVD, Microvessel density.

## DISCUSSION

The anti-proliferative and pro-apoptotic effects of FILIP1L have recently been identified [7–13]. Moreover, FILIP1L expression is decreased in various cancer tissues [14–18], suggesting that FILIP1L suppresses cancer development and progression.

Cell migration and invasion play crucial roles in various physiological processes, including embryonic development, angiogenesis, tissue repair, and immune response, all of which are deregulated in cancer cells [19, 20]. Here, we found that FILIP1L knockdown enhanced, while FILIP1L overexpression suppressed, human colorectal tumor cell migration and invasion. MMP family proteolytic enzymes are critical for extracellular matrix remodeling, and degradation of the extracellular matrix is considered a prerequisite for cancer invasion and metastasis [21, 22]. In a previous study using ovarian cancer cell lines and ovarian orthotopic tumor models, FILIP1L decreased MMP-3, -7, and -9 levels and inhibited cancer cell migration and invasion both *in vitro* and *in vivo* [16]. Here, MMP-2 and -9 levels increased after FILIP1L knockdown, and MMP-2, but not MMP-9, level decreased after FILIP1L overexpression.

Apoptotic cell death results in the orderly and efficient removal of damaged cells, and deregulation of apoptosis contributes to development, progression, and treatment resistance in cancer [23, 24]. Here, apoptosis was inhibited and cell cycle progression increased after FILIP1L knockdown in human colorectal cancer cells. In contrast, both apoptosis and cell cycle arrest increased after FILIP1L overexpression. Additionally, levels of cleaved caspase-3, -7, and -9, the main enzymes in the apoptotic signaling pathway, decreased after FILIP1L knockdown and increased following FILIP1L overexpression. These results suggest that FILIP1L inhibits invasiveness and oncogenesis in human colorectal cancer cells by inhibiting cell migration and invasion and promoting apoptosis and cell cycle arrest.

Angiogenesis is an integral component of progression and metastasis in many cancers, including colorectal cancer [25, 26]. Moreover, tumor angiogenesis as quantified by MVD is a significant negative prognostic factor in human colorectal cancer [27]. A previous study found that FILIP1L protein levels were up-regulated in endothelial cells treated with an angiogenic inhibitor [12]. In addition, administration of truncated mutant FILIP1L inhibited growth in human malignant melanoma tumor xenografts and serous cystadenocarcinoma cells by suppressing angiogenesis [12, 13]. We therefore performed *in vitro* assays to assess the impact of FILIP1L expression on angiogenesis in human colorectal cancer cells. FILIP1L knockdown increased angiogenesis and VEGF-A and HIF-1α levels and decreased angiostatin level. In contrast, FILIP1L overexpression decreased angiogenesis and VEGF-A and -D levels and increased angiostatin and endostatin levels. These results indicate that FILIP1L inhibits angiogenesis, at least in part, by inhibiting the activity of angiogenic inducers and promoting the activity of angiogenic inhibitors in human colorectal cancer.

Next, we examined whether FILIP1L affects the activation of intracellular signaling pathways involved in oncogenic processes, including apoptosis, proliferation, and angiogenesis, in colorectal cancer cells. FILIP1L knockdown increased level of phosphorylated Akt and GSK-3β and decreased levels of phosphorylated β-catenin. In contrast, FILIP1L overexpression increased phosphorylated β-catenin level and decreased phosphorylated Akt and GSK-3β levels. A previous study showed that FILIP1L inhibited cancer cell invasion and metastasis by blocking WNT/β-catenin signaling pathway activity [16]. GSK-3β, which mediates Akt signaling and prevents β-catenin phosphorylation and degradation, is aberrantly activated in many types of cancer [28, 29]. In addition, Akt/GSK-3β/β-catenin signaling pathway activity promotes cell motility, cell survival, angiogenesis, and carcinogenesis in colorectal cancer [30, 31]. These results indicate that FILIP1L may suppress tumor growth by inactivating the Akt/GSK-3β/β-catenin signaling pathway in colorectal cancer.

We also evaluated FILIP1L expression in colorectal cancer patient samples and examined associations between FILIP1L expression and patient prognoses. FILIP1L expression, which was lower in colorectal cancer tissues than in normal colorectal mucosa, was associated with reductions in tumor size, cell differentiation, lymphovascular invasion, cancer stage, invasion depth, and lymph node metastasis, and with longer overall survival. In addition, negative FILIP1L expression was independently associated with poor survival in multivariate analysis. These results suggest that FILIP1L may play a key role in colorectal carcinogenesis and serve as a useful indicator of prognosis in colorectal cancer patients.

Finally, we evaluated associations between FILIP1L expression and human colorectal cancer cell apoptosis, proliferation, and angiogenesis to confirm our *in vitro* results. The mean KI and MVD values were lower in FILIP1L-positive tumors than in FILIP1L-negative tumors. However, AI did not differ depending on FILIP1L expression. A previous study demonstrated that targeted expression of truncated mutant FILIP1L inhibited tumor growth by suppressing cell proliferation and angiogenesis in an ovarian cancer xenograft model [13]. These results confirm that FILIP1L inhibits tumor cell progression and angiogenesis *in vivo* as well as *in vitro*.

Taken together, our results indicate that FILIP1L inhibits progression in colorectal cancer by inhibiting tumor cell proliferation and angiogenesis.

## MATERIALS AND METHODS

### Cell culture

The SW480, DLD1, DKO1, HCT116, HT29, and COLO205 human colorectal carcinoma cell lines were cultured in DMEM (Gibco, Grand Island, NY, USA) supplemented with 10% (v/v) fetal bovine serum (FBS; Gibco) and 1% (v/v) penicillin/streptomycin. HUVECs were purchased from Lonza (Walkersville, MD, USA) and maintained in EBM^™^-2 medium supplemented with EGM^™^-2 Single Quotes^™^ kit (Lonza). All cells were maintained at 37°C in a humidified incubator (Sanyo, Panasonic Healthcare Company, Wood Dale, IL, USA) with 5% CO_2_. To obtain CM for tube formation and Matrigel invasion assays, transfected cells were incubated in serum free medium for 1 day.

### Gene transfection

FILIP1L siRNA (CAGUCAUCAAUGGUCAGUU-dTdT) and scrambled siRNA were purchased from Santa Cruz Biotechnology (Santa Cruz, CA, USA) and Qiagen (MD, USA), respectively. FILIP1LΔC103 (amino acid 1-790) cDNA was cloned into the pcDNA6-myc vector (Invitrogen, Carlsbad, CA, USA) as described previously [12]. After cleavage with restriction endonuclease and identification, the FILIP1L constructs were verified by DNA sequencing. COLO205 and HCT116 cells were seeded evenly, grown to 50–80% confluency, then transiently transfected with FILIP1L siRNA and constructs using Lipofectamine 2000 transfection reagent (Invitrogen) according to the manufacturer's instructions.

### Western blot

Following gene transfection, cells were collected and lysed in ice-cold RIPA extraction solution (Thermo, Rockford, IL, USA). Cell proteins were separated by 8–15% SDS-PAGE and transferred to PVDF membranes using a Mini Trans-Blot Cell and System (Bio-Rad, Hercules, CA). The membranes were blocked with nonfat milk and incubated overnight with primary antibodies diluted in TBST at 4°C. The following primary antibodies were used: FILIP1L, angiostatin, endostatin, and matrix metalloproteinases (MMP)-2 and -9 (Abcam, Cambridge, UK); phospho-Akt (Ser473), Akt, phospho-β-catenin (Ser33/37/Thr41), β-catenin, phospho-glycogen synthase kinase 3 beta (GSK-3β) (Ser9), GSK-3β, cleaved caspase-3, -7, -9, and hypoxia-inducible factor-1α (HIF-1α) (Cell Signaling, Danvers, MA, USA); and vascular endothelial growth factor (VEGF)-A and -D, and GAPDH (Santa Cruz Biotechnology). The membranes were developed using an ECL reagent (Amersham, Arlington Heights, IL, USA). Immunoblots were quantified using Multi-Gauge software (ver 3.0, Fujifilm, Tokyo, Japan).

### Wound healing and transwell invasion assays

Cell migration was evaluated with a wound healing assay using Culture-Inserts (Ibidi, Regensburg, Germany). Transfected COLO205 and HCT116 cells were plated into Culture-Inserts and a wound gap was created by removing inserts after 24 h of incubation. Wounded monolayers were photographed at 0 and 72 h post-wounding under an inverted microscope. Wound closure was normalized to 1 cm at three random sites. Cell invasiveness was determined using the transwell invasion assay. Transfected COLO205 and HCT116 cells were plated into transwell upper chambers coated with gelatin. 400 μL of 0.2% BSA medium containing fibronectin (10 μg/mL) as a chemoattractant was added to the lower chamber. After 24 h of incubation, invaded cells that had reached the bottom surface were fixed with 70% ethanol and stained with Hemacolor^®^ Rapid staining solution (Merck Millipore, Darmstadt, Germany). Cells remaining in the upper chambers were removed with a cotton swab. Cells on the bottom surface were stained and counted under a light microscope.

### Flow cytometric analysis of apoptosis

A FACSCalibur flow cytometer (Becton Dickinson, San Jose, CA, USA) was used to analyze numbers of apoptotic cells with sub-G1 DNA and annexin-V staining. Sub-G1 DNA content proportions were determined using propidium iodide (PI) staining. Cells were fixed in 1 U/mL of RNase A (DNase free) and 10 μg/mL of propidium iodide (Sigma, St. Louis, MO. USA) overnight in the dark at room temperature. For annexin-V staining, living cells were rinsed in phosphate-buffered saline (PBS) and incubated with 7-AAD and annexin V-APC (BD Biosciences, San Diego, CA, USA).

### *In vitro* endothelial tube formation and matrigel invasion assays

The *in vitro* endothelial tube formation assay was performed using Geltrex^™^ reduced growth factor basement membrane matrix (Invitrogen). Briefly, HUVECs suspended in CM were plated on a culture plate coated with Geltrex^™^ matrix and incubated at 37°C in a 5% CO_2_ atmosphere overnight. Each culture was photographed at x 100 magnification using a camera connected to an inverted microscope. The WIMtube image analysis platform (WIMASIS GmbH, Munich, Germany) was used to quantify total tube length. Matrigel invasion assays were performed using transwell filter chambers (8-μM pores, Corning Inc., NY, USA). HUVECs suspended in EGM^®^-2 MV Single Quotes^®^ media were inoculated into upper transwell chambers coated with Matrigel (BD Bioscience). The lower chamber was filled with prepared CM. Invaded HUVECs on the bottom surfaces of the upper chambers were stained with Hemacolor^®^ Rapid staining solution (Merck Millipore), and cells remaining in the upper chambers were removed with a cotton swab. Stained cells were observed under a light microscope and counted in 5 selected fields.

### Patients and tissue samples

354 paraffin-embedded advanced CRC specimens were selected from patients undergoing surgery for colorectal cancer at the Chonnam National University Hwasun Hospital (Jeonnam, Korea) between July 2004 and June 2006. None of the patients had received preoperative radiotherapy or chemotherapy. The Institutional Review Board of the Chonnam National University Hwasun Hospital (CNUHH-2016-117) approved sample use. Tumor-node-metastasis (TNM) stages were assigned using the American Joint Committee on Cancer (AJCC) criteria [32]. Overall survival was calculated from the date of the initial surgery until the follow-up on December 31, 2012.

### Immunohistochemistry and evaluation of FILIP1L expression

Sections were deparaffinized in xylene and rehydrated with graded ethanol solution (100–70%). Antigen retrieval was performed by boiling in citrate buffer (pH 6.0, Dako, Carpentaria, CA, USA), and endogenous peroxidase activity was blocked with peroxidase-blocking solution (Dako). Non-specific reactivity was blocked with DAKO^®^ Protein Block Serum-Free solution (Dako). Sections were incubated with primary anti-FILIP1L (Abcam), -Ki-67 (Dakopatts, Glostrup, Denmark), and -CD34 (Abcam) antibodies. Bound antibody was visualized with the DakoReal^™^ Envision HRP/DAB detection system (Dako). Nuclear counterstaining was performed with hematoxylin (Sigma-Aldrich, St. Louis, MO. USA). Stained tissues were viewed and photographed under a light microscope. Immunohistochemical staining was assessed by two independent pathologists without knowledge of patient clinical outcome data. FILIP1L staining intensity was scored as follows: 0, no staining; 1, weak; 2, moderate; 3, strong. Percentage of cells stained was also scored (1: 0%–25%; 2: 26%–50%; 3: 51%–75%; 4: > 75%). The final score for each sample was calculated by multiplying the staining intensity score by the percentage score. Final scores for the 354 tumor samples ranged from 0.0 to 12.0 with a mean of 7.5. The mean final score value for each sample served as the cut-off point for categorization of the 354 tumor samples into positive and negative expression groups; samples with a final score ≥ 8 were designated positive for FILIP1L expression.

### Assessment of apoptosis and tumor cell proliferation

Tumor cell apoptosis was detected using the terminal deoxynucleotidyl transferase-mediated dUTP nick-end labeling (TUNEL) technology system (Promega, Madison, MA, USA) according to the manufacturer's instructions. Briefly, the tissues were deparaffinized, rehydrated through a graded alcohol series, and incubated in permeabilization solution. Labeling was performed by adding the terminal deoxynucleotide transferase enzyme (TdT) reaction mix to tissue sections mounted on slides. After washing, the slides were incubated with the enzyme substrate 3,3-diaminobenzidine (DAB) for color development, which was used to localize labeled cells. The AI was calculated as the number of TUNEL-positive cells per 1000 tumor cell nuclei. Tumor cell proliferation was visualized by immunostaining with the Ki-67 antibody. Nuclei immunostained with Ki-67 antibody were considered positive. KI was defined as the number of Ki-67-positive nuclei per 1000 tumor cell nuclei.

### Assessment of microvessel density

MVD was measured by quantifying the average number of CD34-positive vessels. Stained slides were examined under low-power light microscopy, and locations with the highest density of stained vessels were identified as ‘hot spots’. Three ‘hot spots’ per case were then counted under higher magnification (×200).

### Statistical analysis

Statistical Package for the Social Sciences (SPSS) version 20.0 software (IBM Corporation, Armonk, NY, USA) was used to conduct statistical analyses. A χ^2^ test was used to analyze the association between FILIP1L expression and clinicopathological parameters. Survival rates were calculated using the Kaplan-Meier method, and the statistical significance of differences was examined using the log-rank test. Multivariate analysis was conducted using Cox proportional hazards regression model. Student's *t* tests were used for comparisons between two groups. Each experiment was repeated at least three times. *P* < 0.05 was considered statistically significant.
